# The Road not Taken: Less Traveled Roads from the TGN to the Plasma Membrane

**DOI:** 10.3390/membranes5010084

**Published:** 2015-03-10

**Authors:** Anne Spang

**Affiliations:** Biozentrum: Growth & Development, University of Basel, Klingelbergstrasse 70, CH-4056 Basel, Switzerland; E-Mail: anne.spang@unibas.ch; Tel.: +49-61-267-2380; Fax: +49-61-267-2148

**Keywords:** membrane transport, vesicle transport, *trans*-Golgi network, exocytosis, secretion, adaptor complex, small GTPase

## Abstract

The trans-Golgi network functions in the distribution of cargo into different transport vesicles that are destined to endosomes, lysosomes and the plasma membrane. Over the years, it has become clear that more than one transport pathway promotes plasma membrane localization of proteins. In spite of the importance of temporal and spatial control of protein localization at the plasma membrane, the regulation of sorting into and the formation of different transport containers are still poorly understood. In this review different transport pathways, with a special emphasis on exomer-dependent transport, and concepts of regulation and sorting at the TGN are discussed.

## 1. Introduction

Cells communicate with their environment through the secretion of factors that are recognized by neighboring cells by receptors that are present in the plasma membrane. Both factors in this communication scheme are inserted first into the ER membrane and transported along the secretory pathway to the plasma membrane where the signaling factor is released into the environment and the receptor is anchored in the plasma membrane. Examples of such communications include certain growth factors (such as EGF; epidermal growth factor) and their receptors (like EGFR; EGF receptor) [1]. But in addition to signaling receptors, the plasma membrane also contains transporter and channels that are essential in the regulation of ion homeostasis and the nutrient state of the cell [2,3,4]. In neurons, neurotransmitters are released from one cell at the synapse and taken up by another contacting neuron [5]. In addition, there are structural proteins that assemble the cell walls of fungi and plants, and the extracellular matrix in metazoans [6,7]. Adhesion molecules present at the plasma membrane allow stable contacts between cells, as for example at neuronal synapses, or they enable cells to form an organ as in the case of epithelial and endothelial cells [8,9]. All these proteins essential for cellular function are brought to the plasma membrane by the secretory pathway. Most proteins present at the plasma membrane are glycosylated. Glycosylation is initiated in the endoplasmic reticulum and extended in the Golgi apparatus. Like plasma membrane proteins, Golgi-resident proteins reach their compartment by the secretory pathway. All key components of the secretory pathway are essential for life; mutations in those components—if not lethal—cause severe diseases. Although in the last 30 years most of the general machinery and key components have been identified, we still understand very little about the regulation of traffic along the secretory pathway. In particular how distribution is regulated at the major sorting stations—early endosomes and the *trans*-Golgi network—is still not entirely clear. Moreover, how post-translational modifications impact the activity of key components, such as small GTPases, coats and adaptors, to tune the transport pathways according to the cellular state, remains largely elusive.

## 2. Traffic along the Secretory Pathway

### 2.1. Getting to the trans-Golgi

**Figure 1:**
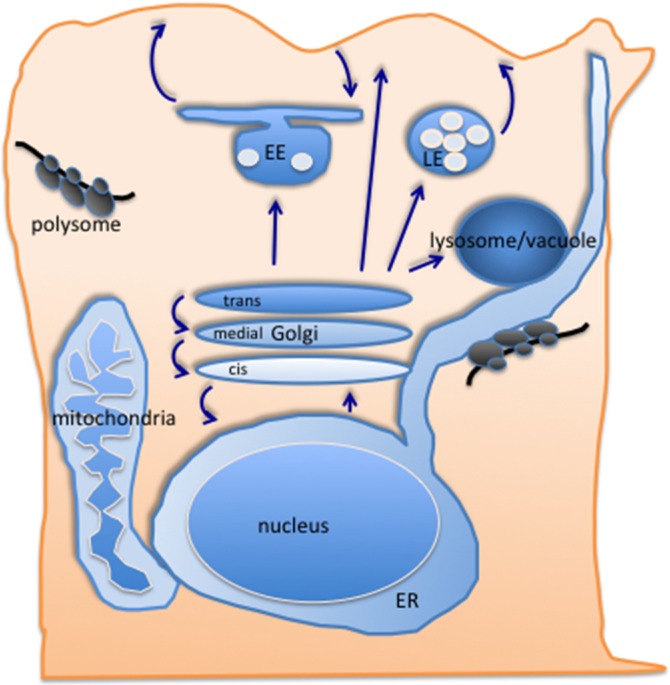
Schematic drawing of the secretory pathway in eukaryotic cells. ER; endoplasmic reticulum, EE; early endosome, LE; late endosome.

Proteins destined for secretion or residence in membrane-bound organelle along the exo- and endocytic pathways are synthesized into the lumen of the endoplasmic reticulum (ER) (Figure 1). There, protein folding occurs and most proteins acquire at least one N-glycan. This modification serves as a beacon for the quality of protein folding, as defined by the burial of hydrophobic residues within the protein structure. It is worthwhile noting that the quality of the fold is assessed in the ER, which should not be confused with functionality of the protein. A protein can attain an acceptable fold but nevertheless remain non-functional; a prominent example is the prion protein PrP [10]. Once a protein is deemed properly folded, it exits the ER through vesicles that are generated by the COPII coat. The inclusion into COPII vesicles is mediated through either direct interaction with the Sec23/24 coat component or a cargo receptor that in turn specifically binds to the same COPII subunits [11]. In some, and perhaps most cases, this interaction also necessitates the presence of the active form of the small GTPase Sar1. The COPII vesicle is released from the ER by a mechanism the final stage of which still remains rather elusive. In most cells, the COPII vesicles are relatively short lived. They are formed at ER exit sites, which are in close proximity to the Golgi apparatus, the recipient organelle of COPII vesicles. In mammalian cells with a juxtanuclear extended Golgi structure, this COPII vesicle receiver is the ERGIC (ER-Golgi intermediate compartment). In other cell types, with a less elaborate central Golgi apparatus, numerous Golgi mini stacks exist, and the *cis*-compartment (the one which is closest to the ER) is only just over 100 nm away from ER exit sites (ERES), as demonstrated by elegant electron microscopy and high resolution light microscopy studies [12,13,14]. These findings are particularly exciting and interesting, considering that a typical COPII vesicle is about 70 nm in diameter [15]. Given that larger cargoes as chylomicron or collagen also need to be transferred from the ER to the Golgi [16,17,18,19,20], it is tempting to speculate that a bridging mechanism might exist, in which COPII would act as cargo recruiter and scaffolder for the bridge through which super-size cargo (too large to be accepted into a normal COPII vesicles) would be transported to the next compartment of the secretory pathway. At least in yeast, Golgi elements appear to contact ERES, causing COPII cages to collapse, and cargo would be transferred [13]. Although this is a fascinating unsolved problem in membrane traffic, it is not the focus of this review and I will leave it to others to discuss this issue in more detail.

Most cargoes will exit the ER through COPII vesicles and will arrive at the *cis*-side of the Golgi apparatus. There, a second function of the N-glycan becomes apparent. This structure is reused to expand the structural and functional repertoire of proteins. Other sugars are added onto the core N-glycan structure by sugar transferases such as glycosyltransferases that are distributed in a particular order through the different Golgi compartments [21]. It is thought that the order in which the sugar transferases encounter a protein is one of the main determinants of the sugar tree structure the protein will acquire during passage through the Golgi. However, not each protein that was initially N-glycosylated in the ER will be modified in exactly the same way in the Golgi apparatus. Thus, there must be something else besides enzyme localization determining the final sugar structure of a particular protein. Supporting this notion, increasing evidence imply that proteins do not all travel at the same rate through the Golgi stacks [22], suggesting that there is more than just one way to get from the *cis*- to the *trans*-side of the Golgi [23,24]. This is another area, which is highly controversial and highly debated in the field of membrane traffic, but again, not the focus of the review.

### 2.2. Exit from the Golgi—The Conventional Routes

While transport through the Golgi requires a maturation process in which the *cis*-cisterna matures first to a medial and then to a *trans*-cisterna, with retrograde vesicle transport to keep the glycosylation enzymes in the right compartment, the *trans*-Golgi network (TGN) is apparently constantly turned over through retrograde and anterograde cargo container formation [23,25]. The anterograde transport containers seem to be heterogeneous in nature, varying from vesicles to different types of tubes and fenestrated membranes [26,27,28,29]. Formation of the transport carriers depends on the cytoskeleton, with motors pulling tubules from the Golgi as well as lipids, such as diacylglycerol (DAG), that are important for the scission step [30,31,32,33].

**Figure 2:**
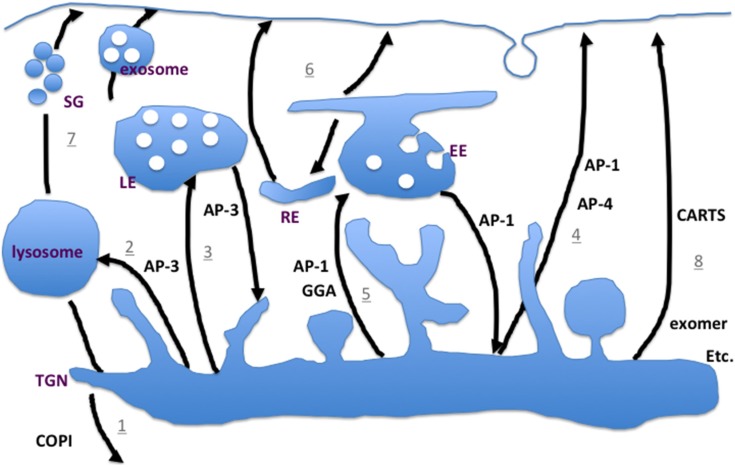
Schematic drawing of the exit pathways from the *trans*-Golgi network (TGN). For more detailed information, see text. SG; secretory granules, EE; early endosomes, LE; late endosomes, RE; recycling endosomes. The numbers refer to the TGN-plasma membrane pathways mentioned in the text.

The cargo at the *trans*-Golgi now has certain possibilities: First it could be sorted into retrograde COPI vesicles, causing its recycling into an earlier Golgi compartment (Figure 2; 1). In the opposite, anterograde direction, cargo can go in a direct way to the lysosome, which depends most likely on AP-3 [34,35,36]. AP-3 is required for transport of a subset of lysosomal proteins in eukaryotes [34,37,38]. While in yeast this transport occurs directly from the TGN to the vacuole (Figure 2; 2), the route is much less clear in metazoans (Figure 2; 3). Convincing evidence exists that there is an AP-3-dependent sorting step at endosomes [39,40,41,42]. However, a role in regulated secretion at the TGN has also been reported [43,44]. Another possibility is to take a direct pathway to the plasma membrane (Figure 2; 4) mediated by AP-1 or AP-4 complex binding. The AP-1 pathway is probably the major export route in different cell types in metazoans [45,46,47,48,49]. While AP-1 and AP-3 bind clathrin, AP-4 dependent transport appears to be independent of clathrin. In addition, cargo can reach the endosomal system at the early (Figure 2; 5) or late endosome and then use recycling pathways (Figure 2; 6) to the plasma membrane to reach their final destination; this appears to be the preferred pathway in yeast. At least a subset of these carriers is also AP-1 covered [50], which is puzzling, as it would suggest that a vesicle formed at one compartment could be targeted to two different locations. Given that the SNAREs for fusion to endosomes are not the same as for fusion with the plasma membrane, potentially another layer of specificity is included. Even more confusing in this context is that AP-1 is also responsible for retrograde transport from early endosomes in yeast and mammalian cells [50,51]. Other export routes from the TGN use GGAs (Golgi-localized, gamma-ear-containing, Arf binding proteins) or epsin-related proteins as clathrin adaptors [52,53,54,55]. It is conceivable that different exit sites on the TGN are present depending on the transport route, which might in part be dependent on the cargo that would accumulate there. Whereas AP-1 and AP-3 recognize at least similar linear peptide sequences, GGAs appear to transport ubiquitylated proteins [56,57,58,59]. Recent data suggest GGAs and AP-1 have overlapping as well as distinct functions. Interestingly, acute removal of AP-1 from the TGN caused also the dissociation of the GGAs from the TGN [50]. The recruitment of AP-1 and GGAs appears to be temporally, and potentially spatially, controlled by the PI4P levels in the Golgi [60]. GGA2 binds directly PI4 kinase in yeast and low PI4P levels would promote rather the separate than overlapping functions of the coats [60]. Thus, some adaptor complexes act on different organelles at the intersection of endocytic and exocytic pathways. Yet another complication is that such exit sites are to some extent defined by the guanine nucleotide exchange factors (GEFs) that control the spatial activation of small GTPases of the Arf/Sar family. The number of ArfGEFs that act at the Golgi is limited and a particular GEF could activate Arf at different sites at the TGN. Therefore the specificity and coordination of GTPase activity, cargo recruitment and coat polymerization must be controlled in part by different factors. Clearly, lipid molecules play an important role in this process [32,61,62,63]. A subset of cargo is sorted in a Ca^2+^-dependent manner [64]. However, these molecules may be insufficient to generate the required variety of exit sites. Finally, secretory granules can be formed at the *trans*-Golgi. They mature during transport to the plasma membrane, dock, and release their cargo after stimulation (Figure 2; 7). One of the best-characterized types of the latter vesicles are secretory vesicles in neurons, which release neurotransmitter at synapses upon Ca^2+^ influx [65]. For all these trafficking pathways most of the basic machinery has been identified and the general process is understood.

### 2.3. Less Studied—But not Less Important—Pathways to the Plasma Membrane

Recently a number of cargoes have been described that reach the plasma membrane in a non-canonical way (Figure 2; 8) [51,66,67]. These cargoes are exported from the TGN in an AP-1 independent manner. For example the receptor Frizzled 6 appears to reach the plasma membrane through a yet unexplored pathway [66]. In addition, Cab45-dependent cargoes and the export of TGN46 and PAUFS are equally independent of known pathways [64,67]. The transport containers for the latter cargoes are referred to as CARTS and their formation depends of protein kinase D (PKD), but the coat and adaptors remain elusive [67].

Another way to regulate trafficking to the plasma membrane has been recently discovered in plants. During cytokinesis, endocytosed cargo from the plasma membrane is diverted from the recycling pathway to the plasma membrane into a different pathway, which will generate a pool of vesicles that undergoes homotypic fusion to generate the novel cell plate [68]. This is of particular interest also to researchers outside of the plant field because the early endosomes and the *trans*-Golgi network are supposed to be the same compartment in plants and other organisms. Therefore, the switching of the pathway may be similar to diverting cargo from one TGN exit pathway to another in yeast and metazoans.

Yet another pathway that in recent years has attracted considerable attention is the formation of exosomes and their fusion to the plasma membrane [69]. Their major purpose might not be to change the proteome that is expressed at the plasma membrane, however, but rather serves for communication and signaling with/to other cells. Since exosomes have apparently a prominent role in a variety of diseases ranging from cancer, to neurological disorders and infections, they have generated great excitement as they may potentially be used as biomarkers for disease states [70]. Exosomes morphologically resemble multivesicular bodies (MVBs) aka late endosomes. It is assumed that they derive from early endosomes [71,72], and that their formation relies at least in part on the same machinery than the one required for the formation of intraluminal vesicles at endosomes [73]. However, the cargo contained in exosomes is to some extent distinct from that of ordinary late endosomes. For example, miRNAs, proteins, and lipids are specifically incorporated into exosomes through so far ill understood mechanisms [72]. For their formation they presumably rely on cargo and membrane contributions from the *trans*-Golgi network as do late endosomes. Hence they could also be viewed as another special type of transport container destined for the plasma membrane.

Over the last couple of years, it has been appreciated that besides the super highways of intracellular transport, less-travelled pathways exist that are important for the spatially and temporally controlled discharge of cargo proteins at the plasma membrane. The best studied of the Golgi exit pathways might be the exomer-dependent transport route from the TGN to the plasma membrane in the yeast *Saccharomyces cerevisiae.* For this pathway, only three different cargoes are yet known, however, they share an impressive amount of regulation that may be a key characteristic of their transport to the plasma membrane.

### 2.4. Exomer-Dependent Transport to the Plasma Membrane

Exomer was discovered as an Arf1 effector complex that is important for the transport of the chitin synthase Chs3 to the plasma membrane in yeast [74,75,76]. It has even been proposed to be a coat for the export of Chs3 to the plasma membrane [76]. In favor of this notion are the findings that exomer requires active Arf1 for membrane association and directly interacts with the cargo molecule [75]. This is in very good analogy to how COPI and COPII coats act. However, while those coats contain the intrinsic ability to deform lipid membranes and bud off coated vesicles from synthetic liposomes [77,78], exomer does not meet this criterion. Therefore, exomer may rather act as a cargo recruiter and a platform provider, through which certain cargoes could be sequestered and then subsequently included into a certain type of vesicles. This model would argue for exomer to behave more like an organizer of special TGN exit sites. Alternatively, exomer may act as an adaptor complex. The adaptor complexes (APs) that capture cargo for clathrin-coated vesicles also lack the intrinsic ability to deform biological membranes. In a fact, a part of the exomer component Chs5, which contains BRCT/fibronectin (FN) domains has a similar fold to AP-2 complex-α chains [79]. There is a complication with the exomer complex as it comes in different flavors. In general, exomer consists of Chs5, and four homologous proteins, the ChAPs (Chs5 and Arf1 binding Proteins). The ChAPs are Chs6, Bud7, Bch1 and Bch2, and they are TPR-containing proteins presumably recognizing exomer-dependent cargo [74,75,76,79,80]. Sanchatjate and Schekman [74] purified exomer with a stoichiometry of 5:4:1:1:1 (Chs5, Bch1, Bud7, Chs6, and Bch2). However, at the same time there were already genetic and biochemical indications that exomer complexes with varying stoichiometries and different ChAP contributions may exist [75]. Indeed, when exomer components were crystallized, it became apparent that the organizational unit of exomer probably consists of a homodimer of Chs5, with each of the Chs5 having one binding site for a ChAP [79]. This is a strong indication that the composition of the exomer complex may change depending on which cargo needs to be transported and is in agreement with previously published data [75]. Hence exomer function would be consistent with being a cargo receptor or an adaptable adaptor complex. Though we still do not know how the exomer complex works precisely and how it selects its client proteins (see below), to date it remains the best understood alternative transport complex for export from the TGN.

### 2.5. Exomer-Dependent Cargoes

What are the cargoes that depend on exomer for TGN export? The first identified cargo was Chs3, which has an interesting cell-cycle dependent localization pattern [81,82,83]. In small (G1/S phases) and large budded (late M phase) cells, Chs3 is present at the bud neck, while in the interim Chs3 is stored in so-called chitosomes, which are identical with the TGN [82,83]. Thus, albeit not being needed in G2 and early M phase, Chs3 is not degraded in the vacuole but rather stored in the TGN, presumably in preparation of a readily releasable pool (see also below). Chs3 is in at least a dimerized, if not oligomerized, state when included into transport vesicles at the TGN [84]. Interestingly, an N-terminal peptide, which is in the same region as the sequences required for dimerization, has been soaked into a Chs5-Chs6 crystal structure, indicating that this peptide recognizes and may contribute to the binding of exomer [85]. This would suggest that the dimerized state of Chs3 might be recognized by exomer. Additionally, Chs3 is palmitoylated, which may be important for the extent of plasma membrane localization [86]. This assumption is difficult to test because palmitoylation is already a prerequisite for ER exit [86]. It is likely that the S-acylation is having an effect on either the residence time at the TGN or the plasma membrane because Chs3 has 6 transmembrane domains, and hence would not need any additional membrane anchor for membrane insertion [84]. Finally, Chs3 steady state localization is also likely relying on its ubiquitylation state. Consistently, it has been shown that Chs3 localization at the bud neck is dependent on constant endocytosis presumably to remove any Chs3 that has escaped the bud neck [82,87]. Thus, Chs3 plasma membrane localization is highly dynamic and equally highly regulated. This type of regulation might be a hallmark for exomer-dependent cargoes.

The second exomer-dependent cargo is Fus1, a factor that uses the exomer-dependent pathway during mating to reach the shmoo tip [88]. It is worth noting that ectopically expressed Fus1 reaches the plasma membrane via an exomer-independent pathway in logarithmically growing cells (unpublished results). These findings are consistent with posttranslational modifications that also in the case of Fus1 may play a role in the determining the exit route from the TGN. The third cargo is the prion-like domain containing protein Pin2. Like Chs3, Pin2 cycles between the TGN and the plasma membrane in a cell-cycle dependent manner [89]. Interestingly, other features are also conserved between Pin2 and Chs3: Both have the same posttranslational modifications such as phosphorylation and ubiquitylation, and palmitoylation has also been predicted for Pin2 [90]. At least ubiquitylation is essential for proper cycling and cell-cycle dependent localization [89].

Moreover, all three exomer-dependent cargoes share a little peculiar trafficking glitch: They all can escape from the TGN to the early endosome, from which they are retrieved in an AP-1 dependent pathway [51,88,89]. Thus besides cycling through the plasma membrane, they all cycle in a short circuit through the endosome. Most importantly, however, is that if retrieval from the endosome is blocked and exomer is absent, Chs3 and Pin2 can still reach the plasma membrane, which in case of Chs3 results in a strong mislocalization phenotype as it is no longer restricted to the bud neck [51,89]. One possible explanation of these results is that exomer-dependent vesicles are not targeted to the same place as AP-1 dependent vesicles that leave the TGN. Unfortunately, exocyst, the tethering complex at the plasma membrane, is nicely localized at the bud tip for most of the cell cycle. However, this might only be the place of the highest concentration of exocyst, and other exocyst complexes may be distributed all over the bud, including the bud neck, where Chs3 would be localized. This possibility is supported by the notion that only early in the yeast cell cycle is bud growth polarized. It switches to isotropic growth later in the cell cycle before secretion is redirected to the bud neck region in preparation of cytokinesis.

Taken together, it appears as though the trafficking of exomer-dependent cargoes is highly regulated to ensure the proper temporal and spatial distribution of these proteins. But what is so special about these cargoes? At least Chs3 and Pin2 are both retained in internal stores rather than being degraded when not needed, suggesting that they would need to be transported to the plasma membrane very quickly in case of an emergency. Perhaps, they sample the environment/the state of the plasma membrane. Chs3 is a chitin synthase that would be able to reinforce chitin production at the bud neck and prevent damage of the cell wall at other places. Pin2 may be a stress detector as it is rapidly endocytosed under a variety of stresses [89]. Indeed both proteins change their steady state localization in response to cell stress [82,89]. For example, under lithium stress, Pin2 is endocytosed and maintained in the TGN through the prion-like domain, and released once the stress has disappeared. For some reason, Pin2 is not simply degraded in response to stress but is kept at the TGN and reappearance at the plasma membrane starts already at 5 min after stress release, which is much faster than what the cell could achieve through new synthesis and transport through the entire secretory pathway. Even under standard growth conditions, neither Chs3 nor Pin2 are degraded after endocytosis but recycle back through the TGN to the plasma membrane. It appears that the highly regulated pathway and the constant recycling through the TGN are hallmarks of exomer-dependent cargoes.

In spite of the similarities in trafficking of the exomer-dependent cargoes, how exomer recognizes cargo remains unclear. There is no single linear peptide motif, which is common between the three exomer cargoes, and also the ChAPs on which they depend on for export are non-identical. Moreover binding and transplantation studies revealed necessary sequences but none of them is sufficient [82,88], indicating that coincident detection or several none linear interaction surfaces ensure proper targeting. This problem circumvents the easy identification of other exomer-dependent cargoes to expand on the roles that have been established on the so far arguably small dataset.

Is this all just a “yeast thing”? Neither Chs5 nor the ChAPs have direct sequence homologues in worms, flies or mammals. However, Chs5 contains FN and BRCT domains and its N-terminus has a similar fold to the α-subunit of adaptor complexes. Thus, one could argue from structural homology that at least Chs5 may have some sort of adaptor function and has been conserved throughout eukaryotic evolution. The ChAPs contain several TPR repeats [80]. However those repeats are found in numerous proteins and are not very telling about homologues in higher eukaryotes. In contrast to Chs5, complete crystal structures are available for the ChAPs Chs6 and Bch1 [79,91]. New bioinformatics tools are available to find structural homologues from a structure deposited in pdb. As more and more structures become available, it is only a question of time until we can find a conserved structure, which might be very revealing. An example that this approach can be useful and even successful is the identification of TSET, a novel adaptor complex in endocytosis [92].

Novel transport machineries will be discovered in metazoans in the not too distant future that will transport only a subset of proteins, but will therefore precisely control the localization of their clients. They will share some structural features with known coats and adaptor complexes and be different at the same time. Exciting times lie ahead in understanding the regulation of intracellular transport ways, both the highways and the small paths.
